# The effect of conditioning stimulus intensity on conditioned pain modulation (CPM) hypoalgesia

**DOI:** 10.1080/24740527.2020.1855972

**Published:** 2021-02-03

**Authors:** Alexia Coulombe-Lévêque, Yannick Tousignant-Laflamme, Guillaume Léonard, Serge Marchand

**Affiliations:** a Centre de recherche sur le vieillissement, Sherbrooke, Quebec, Canada; b School of Rehabilitation, Faculty de medicine and health sciences, Université de Sherbrooke, Sherbrooke, Quebec, Canada; c Centre de recherche du CHUS, Sherbrooke, Quebec, Canada; d Department of Neurosurgery, Faculty of Medicine and Health Sciences, Université de Sherbrooke, Sherbrooke, Quebec, Canada

**Keywords:** pain, pain modulation, conditioned pain modulation, descending inhibition, cold pressor test

## Abstract

**Background**: The magnitude and duration of conditioned pain modulation (CPM) likely depends on the nature and intensity of the conditioning stimulus (CS).

**Aims**: The aim of this study was to measure the effect of CS intensity on the duration of CPM hypoalgesia.

**Methods**: In this single-blind, nonrandomized, repeated measures study, we assessed CPM hypoalgesia in 20 healthy participants following cold pressor tests (CPT) at 7°C and 12°C. The test stimulus, a 60-s heat stimulation, was administered before the CPT and immediately after, and again at 5-min intervals until participants’ pain scores returned to pre-CS levels. Two hypoalgesia thresholds were used to establish return to pre-CS level: within −10/100 of baseline and within −20/100 of baseline.

**Results**: CPM hypoalgesia, when defined as a reduction in pain levels >10/100, did not last longer following the more intense 7°C CPT compared to the 12°C CPT (32 min vs. 20 min, respectively; *P* = 0.06); similar results were obtained when CPM hypoalgesia was defined as a reduction in pain levels of >20/100 (16 min following the 7°C CPT vs. 9 min following the 12°C CPT; *P* = 0.33). The duration of CPM hypoalgesia was significantly longer when the 10/100 threshold was used compared to the 20/100 threshold, regardless of CPT temperature (*P* = 0.008 for the 12°C CPT; *P* < 0.001 for the 7°C CPT).

**Conclusions**: The more intense CS did not induce CPM hypoalgesia of longer duration compared to the less intense CS. The choice of threshold for what constitutes CPM hypoalgesia did have a significant effect on the results.

## RÉSUMÉ

**Contexte**: L’ampleur et la durée de la modulation de la douleur conditionnée (MDC) dépendent probablement de la nature et de l’intensité du stimulus de conditionnement.

**Objectifs**: Le but de cette étude était de mesurer l’effet de l’intensité du stimulus de conditionnement sur la durée de l’hypoalgésie par MDC.

**Méthodes**: Dans cette étude en simple aveugle, non randomisée, à mesures répétées, nous avons évalué l’hypoalgésie par MDC chez 20 participants en bonne santé à la suite de tests au froid à 7 ° C et 12 ° C. Le stimulus du test, une stimulation thermique de 60 secondes, a été administré avant le test au froid et immédiatement après, puis à nouveau à des intervalles de cinq minutes jusqu’à ce que les scores de douleur des participants reviennent aux niveaux antérieurs au stimulus de conditionnement.

Deux seuils d’hypoalgésie ont été utilisés pour établir le retour au niveau antérieur au stimulus de conditionnement : à l’intérieur de - 10 / 100 de la situation de départ et à l’intérieur de - 20 / 100 de la situation de départ.

**Résultats**: L’hypoalgésie par MDC, définie comme une réduction des niveaux de douleur > 10 / 100, n’a pas duré plus longtemps après le test au froid plus intense de 7 ° C que le test au froid de 12 ° C (32 minutes comparativement à 20 minutes, respectivement ; P = 0,06) ; des résultats similaires ont été obtenus lorsque l’hypoalgésie par MDC était définie comme une réduction des niveaux de douleur > 20 / 100 (16 minutes après le test au froid à 7 ° C comparativement à 9 minutes après le test au froid à 12 ° C ; P = 0,33).

La durée de l’hypoalgésie par MDC était significativement plus longue lorsque le seuil 10 / 100 était utilisé comparativement au seuil 20 / 100, quelle que soit la température du test au froid (P = 0,008 pour le test au froid à 12 ° C ; P < 0,001 pour le test au froid à 7 ° C).

**Conclusions**: Le stimulus de conditionnement plus intense n’a pas induit d’hypoalgésie par MDC de plus longue durée comparativement au stimulus de conditionnement moins intense. Le choix du seuil pour ce qui constitue une hypoalgésie par MDC a eu un effet significatif sur les résultats.

## Introduction

Pain perception is modulated by various intrinsic mechanisms, such as the diffuse noxious inhibitory controls described in animal models by Le Bars et al.,^1^ wherein a noxious stimulus induces widespread hypoalgesia. This phenomenon is studied in humans under the umbrella term “conditioned pain modulation” (CPM),^2^ and its dysfunction has been implicated in the development, maintenance, and exacerbation of many chronic pain conditions,^3^ including fibromyalgia, osteoarthritis, irritable bowel syndrome, temporomandibular disorder, and atypical trigeminal neuralgia.^4–8^ CPM response can be hyperalgesic (i.e., resulting in more intense pain sensations) or hypoalgesic (i.e., resulting in milder pain sensations); a hypoalgesic response is more common^3^ and is the focus of the present study.

CPM response can be influenced by a number of biopsychosocial factors (for a review, see Lewis et al.^3^). For instance, men generally show greater CPM hypoalgesia compared to women,^4,9,10^ and patients suffering from chronic pain tend to show milder and shorter CPM hypoalgesia^3^ compared to healthy subjects.^11^ CPM is also influenced by pain catastrophizing^9^ and by expectations of pain arising from suggestions or beliefs.^12–15^

CPM can also be affected by the characteristics of the triggering noxious stimulus, also known as the conditioning stimulus (CS).^16,17^ More specifically, it appears that the *intensity* and/or the *nature* of the CS could affect the *magnitude* and/or the *duration* of the CPM response; however, the relationships between these variables have not been systematically studied, and the evidence on hand is rather sparse and heterogeneous. It does seem that the magnitude of CPM hypoalgesia can be affected both by the intensity of the CS^18–21^ and by its nature (i.e., type of painful stimulus).^22–25^ Indeed, the magnitude of CPM hypoalgesia is generally correlated with CS intensity, where a more intense CS generates more potent CPM hypoalgesia^14^; moreover, a recent study has shown that three different types of CS (cold pressor test [CPT], cuff pressure pain stimulation, and thermode-based cold pain stimulation), all calibrated to induce pain rated at 55 ± 5/100, reduced the intensity of perceived pain by 43%, 25%, and 23%, respectively.^25^ The duration of CPM hypoalgesia has also been investigated, with authors reporting hypoalgesia lasting up to 10 min^26^ and 30 min^26^; unfortunately, these two studies were too heterogeneous to determine whether variations in the duration of CPM hypoalgesia were attributable to CS type, CS intensity, or a combination of both.

The aim of our study was to assess the effect of CS intensity on the duration of CPM hypoalgesia; more specifically, we wanted to determine whether a 7°C CPT (i.e., a cold-water bath) would induce longer-lasting CPM hypoalgesia compared to a 12°C CPT. Our hypothesis was that the more intense CS (7°C CPT) would result in a CPM hypoalgesia of longer duration than the less intense CS.

## Methods

### Participants

We recruited 20 healthy subjects aged 18 to 45 years old, suffering from no known diseases and not regularly taking pain medication. The study had a crossover design, with all participants undergoing the two experimental conditions. We requested that participants not take short term over-the-counter painkillers, such as ibuprofen, during the 24 h leading up to the two experimental visits and that they not ingest more than their usual dose of caffeine^3^ on the day of the experiment (we did not ask them to forgo caffeine entirely, to avoid potential withdrawal headaches). The procedure took place at the Centre de recherche du Centre hospitalier universitaire de Sherbrooke, Sherbrooke, Québec, Canada. Written informed consent was obtained from all participants, and the study was conducted in accordance with the Declaration of Helsinki and was approved by the ethics review board of the Centre de recherche du Centre hospitalier universitaire de Sherbrooke (ethics approval number 05-021-M9).

### Experimental Procedure

#### Testing Sequence and Apparatus

We used a CPM testing procedure inspired by that of Yarnitsky et al.,^2^ described in our previously published studies,^21,27,28^ wherein a test stimulus (TS) is applied before and after a CS. CPM is measured as the difference in pain levels elicited by the TS before and after the CS. The time of day at which participants were tested differed among participants, but for each participant both sessions took place at the same time of day. The TS was generated by a 3 × 3 cm thermode (TSA II, NeuroSensory Analyzer, Medoc Instruments, Durham, NC), applied on the right forearm of participants. Pain perception was assessed with a computerized visual analog scale (CoVAS) with the left boundary identified as *no pain* (score = 0) and the right boundary as *intolerable pain* (score = 100). Pretests were conducted wherein the thermode temperature was gradually increased; participants were instructed to start moving the cursor toward the right when the heat sensation became painful and that it should reach the right boundary when the pain became intolerable. Results from these pretests were used to identify the Pain_50_ temperature (i.e., the temperature evoking a pain sensation rated as 50/100) for every participant, which was used for all formal TS.^21,29,30^ The CS consisted of a CPT at 12°C or 7°C. The CPT is considered a reliable method to activate CPM hypoalgesia^23,31,32^ and has been shown to reduce TS pain ratings by up to 40%.^21^ The 12°C and 7°C temperatures were chosen because they had previously been shown to induce short-term CPM hypoalgesia of different magnitudes.^21^

#### Pre-CS Test Stimulus (T_0_)

Our TS consisted of a noxious heat stimulus generated by the thermode applied for 60 s on the anterior right forearm at the predetermined Pain_50_ temperature. Subjects were told that the thermode temperature could increase, remain stable, or decrease over the course of the stimulation and that they should record their level of pain throughout the test using the CoVAS. In fact, after a constant rise (0.3°C/s) from baseline (32°C) to the predetermined temperature, the thermode temperature remained fixed throughout the TS (60 s total). The average pain score over the 60-s stimulation was computed, and this score (i.e., the pre-CS TS score) was used for comparison with post-CS measurements.

#### Conditioning Stimulus

The CS consisted of a CPT, wherein participants immersed their left arm in cold water for 2 min. The CPT was administered within the minute following the pre-CS TS. The pain levels elicited by the CPT were recorded every 15 s using the CoVAS. Subjects who could not bear to hold their arm in cold water for the full 2 min were automatically given a score of 100/100.^33,34^ Each participant completed the procedure twice: on day 1 with the CPT at 12°C and on day 2 with the CPT at 7°C. Participants, but not researchers, were blinded to CPT temperatures.

#### Post-CS Test Stimulus (T_1_, T_2_, …, T_n_)

The TS was re-administered at regular intervals following the CS, so that the duration of CPM hypoalgesia could be assessed. The first post-CS TS was administered immediately (within 1 min) after the CS and subsequently repeated at 5-min intervals; in other words, establishing time 0 as the end of the CS, post-CS TS were administered at *t* ≈ 0 min, *t* = 5 min, *t* = 10 min, *t* = 15 min, etc., until pain ratings returned within 10/100 of pre-CPT levels for two consecutive measurements (see data analysis for more details). Each post-CS TS was identical to the pre-CS TS, except that the exact location of the thermode was slightly modified for each post-CS TS to avoid sensitization. As with the pre-CS TS, pain ratings were continuously recorded on the CoVAS throughout the duration of the post-CS TS. The pain ratings obtained throughout each TS were averaged to yield a single pain score for each post-CS TS, and this score was used for comparison with the pre-CS TS score.

### Data Analysis

#### Duration of CPM Hypoalgesia

CPM was measured by comparing the levels of pain elicited by the thermode before and after the CS. That is, for each post-CS measurement, we calculated the difference between the pre-CS TS score and the post-CS TS score, such that a positive value indicated a reduction in pain perception (i.e., hypoalgesia). We set the threshold for CPM hypoalgesia at 10/100,^35^ such that participants showing a pain reduction ≥10/100 were considered to show CPM hypoalgesia. A single score below threshold was not sufficient to assume cessation of CPM hypoalgesia; participants with a single subthreshold score were still considered to be showing CPM hypoalgesia provided that they met this threshold again at the subsequent measurement. Participants were no longer considered to show CPM hypoalgesia when their hypoalgesia scores failed to meet the threshold for two consecutive measurements, and the duration of their CPM hypoalgesia was registered as the last sample time when hypoalgesia was observed (for example, if a participant showed hypoalgesia at *t* = 10 min but not at *t* = 15 min or at *t* = 20 min, we considered that CPM hypoalgesia had been present for 10 min). We also re-analyzed the data, using a more conservative threshold for CPM hypoalgesia set at 20/100.

#### Statistical Analysis

Because of the small number of subjects (*n* < 30) and because visual inspection of the histograms did not allow us to assume that the data were normally distributed, all statistical tests used were nonparametric. Sample size was not calculated a priori, because we did not have access to similar studies whose results and effect size could have informed such sample size calculations. The duration of CPM hypoalgesia was defined for each participant as outlined in the section above, and the scores were averaged to obtain a mean duration of CPM hypoalgesia for the two conditions (7°C CPT and 12°C CPT). The averages were compared using the Wilcoxon signed-rank test. The same test was used to compare, within each condition, the average duration of CPM hypoalgesia that was obtained using the two hypoalgesia thresholds (see above). Finally, as part of a post hoc, exploratory analysis, we assessed the correlation between the duration and magnitude of of CPM hypoalgesia (using the magnitude of CPM hypoalgesia immediately post-CS and 5 min post-CS) using Spearman’s rank correlation coefficient. All tests were performed using SPSS (version 17.0 for Windows; SPSS Inc., Chicago, IL). The threshold for statistical significance was set at *P* < 0.05.

## Results

### Participants and Missing Data

Our sample consisted of 20 volunteers (10 men and 10 women), aged 24.8 ± 3.5 years old. One participant only tolerated 90 s of immersion during the 7°C CPT; all others were able to tolerate both CPTs for the whole duration (120 s) of the immersion. The 7°C CPT, which induced an average pain rating of 62 ± 19/100, was, as expected, significantly more painful than the 12°C CPT, which induced an average pain rating of 39 ± 20/100 (*P* < 0.001).

For technological reasons, we were unable to record some post-CS TS pain scores. Altogether, between our 20 participants undergoing the experiment twice and pain ratings being collected every 5 min until they failed to show hypoalgesia for two consecutive measurements, we ended up with a total of 8.7% missing data (35 out of 404 data points). We elected to linearly interpolate the missing data using immediately adjacent scores.

### Duration of CPM Hypoalgesia

**Figure 1.**
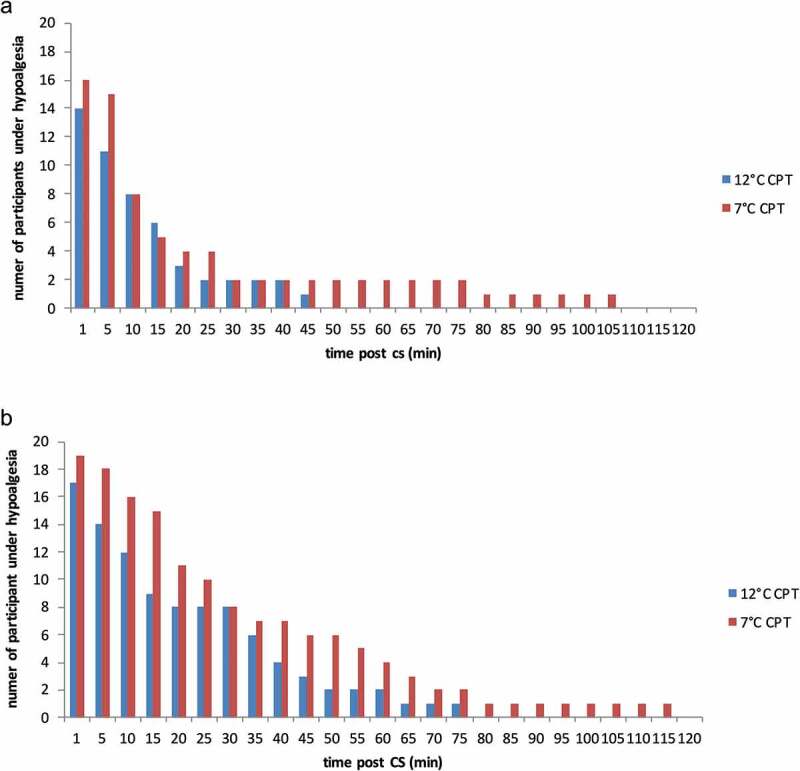
Number of participants exhibiting CPM hypoalgesia at each sampling time following the 12°C and 7°C CPT. Data obtained using the threshold for hypoalgesia of (a) ≥20/100 and (b) ≥10/100. CPT indicates cold pressor test; CPM, conditioned pain modulation

The number of participants showing CPM hypoalgesia at every post-CS TS measurement is shown in Figure 1. The longest duration of hypoalgesia observed in both conditions is also shown in this figure, represented as the last sampling time with at least one participant still showing CPM hypoalgesia. Following the less intense CS (12°C CPT), the longest duration of CPM hypoalgesia observed was 75 min with the ≥10/100 threshold and 45 min with the ≥20/100 threshold; following the more intense CS (7°C CPT), the longest duration of CPM hypoalgesia was 115 and 105 min, respectively, depending on the threshold.

The mean duration of CPM hypoalgesia following the 12°C CPT and the 7°C CPT, obtained using the two thresholds (≥10 and ≥20), is reported in Table 1. CPM hypoalgesia duration appeared to be more than 50% longer following the 7°C CPT compared to the 12°C CPT, but this difference was not statistically significant for either threshold (*P* = 0.06 and *P* = 0.33, respectively). The difference in duration obtained with the two thresholds, on the other hand, was statistically significant (*P* = 0.008 for the 12°C CPT and *P* = 0.001 for the 7°C CPT). In other words, though no difference was observed in the duration of CPM hypoalgesia following the two different CPTs, the choice of threshold had a significant effect on the results we obtained.

**Table 1.** Mean duration of hypoalgesia following the 12°C and 7°C CPT

	Mean duration of hypoalgesia (minutes)		
	12°C CPT	7°C CPT	*P* value
Threshold: 20	9	16	0.332
Threshold: 10	20	32	0.061
*P* value	0.008	0.001	

Duration is in minutes. Results obtained from analysis using the two thresholds for hypoalgesia (pain reduction of ≥20/100 and ≥10/100) are presented.

CPT indicates cold pressor test.

We assessed the correlation between the magnitude of CPM hypoalgesia (at *t* ≈ 0 min post-CS and *t* = 5 min post-CS) and the duration of CPM hypoalgesia for both conditions (12°C CPT and 7°C CPT), under both thresholds (≥10 and ≥20; Table 2). With a single exception (12°C CPT under the ≥20 threshold only), the magnitude of CPM hypoalgesia at *t* ≈ 0 min post-CS showed no correlation with CPM duration (all *P*s > 0.5; all Spearman’s ρ < 0.15). In contrast, the magnitude of CPM hypoalgesia at *t* = 5 min post-CS was significantly correlated with the duration of CPM hypoalgesia, for both conditions and under both thresholds (0.65 ≤ all Spearman’s ρ ≤ 0.75; all *P*s < 0.002). Scatterplots showing individual data for each of the eight correlations calculated (threshold: 10/100 or 20/100; CPT: 7°C or 12°C; time post-CS: ≈ 0 min or 5 min) are presented in Supplementary Figure 1a–h.

**Table 2.** Correlation between magnitude and duration of CPM hypoalgesia

Time post-CS		CPT	Threshold	Spearman Rs	*p* value
t ≈ 0 min	12°C		≥ 10	0.15	0.53
			≥ 20	0.5	0.02
	7°C		≥ 10	0.07	0.77
			≥ 20	0.11	0.67
t = 5 min	12°C		≥ 10	0.73	<0.001
			≥ 20	0.68	0.001
	7°C		≥ 10	0.65	0.002
			≥ 20	0.72	<0.001

The correlations were calculated for both conditions (12°C CPT and 7°C CPT), for all combinations of CPM hypoalgesia scores (taken immediately post-CS or at *t* = 5 min post-CS) and duration scores (calculated with a ≥10 or ≥20 hypoalgesia threshold).

CPM indicates conditioned pain modulation; CS, conditioning stimulus; CPT, cold pressor test.

## Discussion

Our results show that the CPM hypoalgesia induced by the 7°C CPT did not outlast the CPM hypoalgesia induced by the 12°C CPT, which contradicts our initial hypothesis. However, these findings should not be regarded as strong proof that the duration of CPM hypoalgesia is entirely independent of CS intensity, for two reasons. First, it is possible that our lack of statistical significance is attributable to our low sample size and resulting low power. Indeed, our ability to identify a difference in the duration of hypoalgesia (calculated as 1 − β) was 19% when the 20/100 threshold was used and 40% when the 10/100 threshold was used, very much below the standard 80%. Indeed, the difference between the average duration of CPM hypoalgesia following the 12°C CPT and the 7°C CPT is substantial (20 min vs. 32 min with a ≥10/100 threshold), and though the associated *P* value is not statistically significant (*P* = 0.06), it is close enough to the significance threshold that a larger sample may have yielded significant results. We therefore recommend that future researchers use our results to calculate an appropriate sample size to have enough power to detect an effect, if there is one to be found. Moreover, we failed to randomize the two experimental conditions, which may have resulted in an order effect (we did, however, ensure that the participants were naïve to the CPT temperatures). As such, though our results do not support the hypothesis that CS intensity affects CPM hypoalgesia duration, they do not strongly refute it either. Nevertheless, as they stand, our results can be of some comfort to future CPM study participants: indeed, it appears that undergoing the painful 7°C CPT might not be required: the 12°C CPT seems just as apt at triggering CPM.

As we have previously mentioned, various studies on CPM have reported different CPM durations,^26,36^ which suggests that CPM is not an all-or-nothing phenomenon and that some variables (including characteristics of the participants, such as age, sex, etc., and characteristics of the CS^3,26,36^) affect CPM duration. However, CPM duration remains, in our opinion, understudied: to the best of our knowledge, we are the first team to systematically study the effect of CS intensity on the duration of CPM hypoalgesia, despite the fact that the duration of CPM hypoalgesia is a variable relevant to both fundamental and clinical research. Indeed, understanding the factors influencing the variability in the duration of CPM hypoalgesia could help shed light on the neurophysiological mechanisms underlying CPM hypoalgesia; a better understanding of normal processes would in turn allow for a better understanding of their dysfunction, such as is seen in chronic pain conditions. For example, though it has been established that many patients suffering from chronic pain show milder CPM hypoalgesia,^3–8^ the duration of CPM hypoalgesia has seldom been studied in this population, and it is entirely plausible that they exhibit CPM hypoalgesia that is not only milder but also of shorter duration, which could in turn partly explain their clinical features. Of course, these are only speculations, and further studies should be undertaken to properly assess the effect of CS intensity on the duration of CPM hypoalgesia. Interestingly, our results show a moderate correlation between the magnitude and duration of CPM hypoalgesia 5 min post-CS but not immediately post-CS. The implications of these results are twofold: first, the correlation between the duration and magnitude of CPM hypoalgesia 5 min post-CS indicates that clinicians and researchers may be able to infer valuable (if not perfectly accurate) information regarding their patient’s or participant’s CPM hypoalgesia duration simply by measuring its magnitude, thereby allowing them to forgo the tedious and resource-consuming 2-h-long testing session that would otherwise be requiered to assess CPM duration. Second, the absence of correlation obtained between the magnitude and duration of CPM hypoalgesia when CPM magnitude is measured immediately post-CS suggests that CPM response is initially noisy and stabilizes over the course of a few minutes post-CS. The source of this initial fluctuation has yet to be determined: it could stem from a residual distraction of having one’s arm dunked in cold water, or from the time required for the physiological processes underlying CPM to get fully underway, or from something else entirely. In any case, it follows from our results that waiting a few minutes post-CS to measure CPM may yield more reliable information regarding CPM response compared to measuring CPM immediately following the CS. However, these results should be taken with caution, because our small sample size and the relatively high proportion of participants with a small and short-lived CPM response may increase the extent to which these correlations are driven by outliers.

A incidental finding or our study concerns the choice of hypoalgesia threshold (10/100 vs. 20/100), which turned out to have a statistically significant effect on the results. And though this is entirely intuitive, the ramifications are worth considering. Indeed, many studies on pain require that a significance threshold be set. The typical conservative threshold of 20/100 is well accepted for clinical significance,^37^ although some authors have argued for the use of a 15/100 threshold in certain populations, notably the elderly,^38^ and others advocate the need for a rather more conservative threshold, such as 30% or even 50%.^39^ However, it may be that different thresholds are appropriate depending on the research context. Indeed, if one’s goal is to assess whether an intervention can decrease pain levels in a patient population in a way that is clinically meaningful and outweighs the costs of treatment, then perhaps a threshold of 20/100 or more would be preferable. However, if one’s goal is to gain a better understanding of the neurophysiological mechanisms underlying pain modulation, perhaps a pain reduction of 10/100 is large enough to denote a physiologically meaningful effect. Researchers should therefore choose their threshold carefully, keeping in mind both the context of their study and the dramatic effect that the choice of threshold can have on effect size and the ability to detect changes.

## Conclusion

Our results do not support the hypothesis that increasing CS intensity increases the duration of CPM hypoalgesia. CPM hypoalgesia, when defined as a reduction of pain levels of 10/100 or more, lasted on average 32 min following the 7°C CPT and 20 min following the 12°C CPT, with no statistical difference in duration between the two conditions. However, readers should keep in mind that our sample size was small and our conditions were not randomized; additional, high-powered research will be required to draw robust conclusions. Our results also show that the choice of threshold has a nonnegligible impact on results, which highlights the importance of choosing this threshold carefully, keeping in mind the study context (clinical vs. mechanistic).

## Supplementary Material

Supplemental Material
