# Supplementary material for: The effect of conditioning stimulus intensity on conditioned pain modulation (CPM) hypoalgesia
Source: Can J Pain. 2021 Feb 3;5(1):22–9. doi: 10.1080/24740527.2020.1855972 (PMC7951153; doi:10.1080/24740527.2020.1855972)
Supplement: Supplemental Material [file UCJP_A_1855972_SM2887.docx]

*[Supplementary figure 1a-h]*

**Figure legend:**
Supplementary Fig 1a-h. Scatterplots of the correlation between initial CPM score (delta between pre-CS and post-CS TS pain scores, wherein a positive number represents a reduction in pain levels) and CPM hypoalgesia duration. Fig 1a-d depict the correlation with CPM scores taken at t≈0 min post-CS, for both conditions (7°C CPT and 12°C CPT) and under both thresholds (10/100 and 20/100). Fig 1e-h similarly depict the correlation with CPM scores taken at t=5 mins post-CS.

CS: conditioning stimulus; CPM: conditioned pain modulation; CPT: cold pressor test
